# Multistage CBAM-enhanced deep learning for three-class classification of radiographically derived bone mass status labels on knee radiographs

**DOI:** 10.3389/fmed.2026.1960438

**Published:** 2026-10-15

**Authors:** Songjia Li, Jing Lv, Tao Fang

**Affiliations:** 1 School of Acupuncture and Orthopedics, Hubei University of Chinese Medicine, Wuhan, Hubei, China; 2 Orthopedics and Traumatology, Xianning Hospital of Traditional Chinese Medicine, Xianning, Hubei, China

**Keywords:** CBAM, deep learning, grad-CAM, knee radiography, opportunistic screening, osteoporosis

## Abstract

**Objective:**

To develop and validate a deep learning model incorporating multistage convolutional block attention modules (CBAMs) for classifying the radiographically derived bone-mass labels (normal bone mass, osteopenia, and osteoporosis) of a public knee radiograph dataset, and to investigate the potential of knee radiography for opportunistic screening of abnormal bone mass.

**Methods:**

This study used the publicly available Multi-Class Knee Osteoporosis x-Ray Dataset, which was released on Kaggle and compiled from multiple publicly accessible knee radiograph sources. The class labels (normal bone mass, osteopenia, and osteoporosis) were assigned by the dataset provider on the basis of radiographic features and were not verified against dual-energy x-ray absorptiometry (DXA) T-scores; the model therefore classifies the dataset's radiographic labels rather than DXA-defined bone mass status. After assessing image integrity, label consistency, and data usability, a total of 1,947 knee radiographs were included, comprising 780 images labeled as normal bone mass, 374 as osteopenia, and 793 as osteoporosis. The dataset was stratified into training, validation, and test sets at a ratio of 70:20:10. All images were resized to 224 × 224 pixels and normalized using the ImageNet mean and standard deviation. A multi-stage convolutional block attention module (CBAM)-enhanced classification model was developed based on a VGG19-style convolutional neural network and compared with the ResNet50, EfficientNet-B0, DenseNet121, and VGG19 baseline models, as well as several attention-based ablation models. Model performance was evaluated using overall accuracy, class-wise precision, recall, and F1-score, together with confusion matrices and one-vs.-rest receiver operating characteristic curves. Gradient-weighted class activation mapping (Grad-CAM) was further used to visualize and assess the image regions contributing to model predictions.

**Results:**

On the independent test set, the multistage CBAM model achieved the highest overall accuracy of 90.77% (177/195), outperforming VGG19, ResNet50, EfficientNet-B0, DenseNet121, and a reproduced model from the literature. The area under the ROC curve (AUC) was 0.982 for normal bone mass, 0.991 for osteopenia, and 0.981 for osteoporosis. The confusion matrix showed 68/78, 34/38, and 75/79 correctly classified images in the three categories, respectively, with most errors occurring between adjacent bone-mass states. Ablation experiments showed that multistage CBAM outperformed attention-free VGG19, SE-VGG19, ECA-VGG19, and stacked CBAM. Grad-CAM highlighted primarily the distal femur, proximal tibia, and periarticular osseous structures. All results were obtained from a single stratified split and a single random seed (42); because of limited computational resources, repeated runs over multiple seeds and k-fold cross-validation could not be performed.

**Conclusion:**

The multistage CBAM-enhanced model effectively distinguished the radiographically derived bone-mass labels (normal bone mass, osteopenia, and osteoporosis) of this public knee radiograph dataset. Because the labels were not verified against DXA, the model should be regarded as a classifier of the dataset's radiographic labels rather than of DXA-defined bone mass status. It may serve as an adjunctive tool for opportunistic screening of abnormal bone mass and provide imaging-based prompts for subsequent DXA assessment and fracture-risk evaluation, subject to further validation against DXA-derived ground truth, de-duplication and patient-level partitioning, and external data.

## 1 Introduction

Osteoporosis is a systemic skeletal disorder characterized by reduced bone mass, deterioration of bone microarchitecture, and increased fracture risk. Early identification and risk stratification are therefore important for reducing the burden of fragility fractures (1, 2). Dual-energy x-ray absorptiometry (DXA) remains central to bone mineral density assessment and the diagnosis of osteoporosis; however, in routine practice, limited DXA coverage, suboptimal patient adherence, and insufficient screening uptake mean that many individuals with low bone mass or osteoporosis are not identified before sustaining a fracture (3). Current screening recommendations emphasize proactive assessment of osteoporosis risk in older women and postmenopausal women at high risk, underscoring the need to broaden screening coverage. In contrast, conventional radiography is widely used for osteoarticular pain, degenerative disease, trauma, and preoperative assessment, and knee radiographs are particularly common in middle-aged and older adults. Although plain radiography cannot replace DXA for quantitative bone mineral density measurement, it contains potentially informative features, including cortical contours, trabecular texture, grayscale distribution, and local osseous structural changes. In recent years, deep learning has been used to extract features related to abnormal bone mass from hip, lumbar spine, chest, and knee radiographs, suggesting that routine radiographs may provide an entry point for opportunistic osteoporosis screening (4). Nevertheless, most previous studies have focused on binary classification or comparisons of single backbones. Fine-grained discrimination across the continuum from normal bone mass to osteopenia and osteoporosis remains insufficiently studied, and the structural role and interpretability of attention mechanisms in bone mass classification require further clarification (5).

Osteoporosis and osteopenia are highly prevalent in aging populations, and the resulting fragility fractures impose a substantial burden on patients, health systems, and society (1, 6). Because bone loss is clinically silent until a fracture occurs, effective screening depends on identifying at-risk individuals before the first fracture (7). Although DXA remains the reference standard for bone mineral density assessment, its availability is limited in many settings and adherence to screening recommendations is incomplete, so a considerable proportion of individuals with low bone mass remain undiagnosed (8). Routine radiographs obtained for unrelated musculoskeletal complaints therefore represent a low-cost opportunity for opportunistic assessment of bone health (2, 9). Knee radiographs are particularly frequent in middle-aged and older adults, being obtained for pain, degenerative change, trauma, or preoperative planning, and they routinely include the distal femur, proximal tibia, and periarticular trabecular bone, all of which are affected by bone loss.

Against this background, we developed a multistage CBAM-enhanced deep learning model for three-class classification of the radiographically derived bone-mass labels (normal bone mass, osteopenia, and osteoporosis) on routine knee radiographs. Rather than relying solely on a deeper backbone or a single terminal attention module, the proposed network incorporates joint channel-spatial attention at multiple convolutional feature stages to improve selective modeling of osseous texture, grayscale distribution, and local anatomical regions. We further performed comparisons with widely used convolutional backbones, SE/ECA/CBAM ablation experiments, ROC analysis, confusion-matrix analysis, and Grad-CAM visualization to systematically evaluate classification performance, class separability, and spatial attention patterns. The objective was not to replace DXA for definitive diagnosis, but to explore an intelligent adjunctive screening strategy that could be integrated into routine imaging workflows and provide methodological support for identifying individuals with low bone mass, prompting DXA referral, and informing fracture-risk management.

### 1.1 Related work and research gap

Recent deep learning studies have explored opportunistic osteoporosis screening from plain radiographs, including hand (3), hip (10), lumbar spine (11, 12), and knee radiographs (4, 13). Knee radiographs are particularly common in middle-aged and older adults, and several transfer-learning and deep-convolutional approaches have been reported for knee-radiograph-based osteoporosis and osteopenia classification (4, 13, 14), with some studies adopting attention mechanisms or enhanced feature-extraction strategies to improve discrimination (15, 16). Nevertheless, most existing studies addressed binary classification (osteoporosis versus normal) or compared single backbones, and few examined the full three-class continuum from normal bone mass through osteopenia to osteoporosis on knee radiographs. The role of attention mechanism design, in particular attention type, insertion location, and stage multiplicity, in bone mass classification, and the interpretability of such models, remain insufficiently characterized (5). These gaps motivated the present study, which uses a multistage CBAM architecture on the same publicly available knee radiograph dataset to evaluate three-class discrimination and attention design jointly.

Attention mechanisms have become a standard component of medical image analysis because they allow a network to modulate feature responses according to task-relevant evidence rather than treating all spatial locations and channels equally (17, 18). Channel attention recalibrates the relative importance of feature maps, whereas spatial attention emphasizes informative regions, and combining the two has improved performance across several diagnostic tasks (15, 16). In musculoskeletal imaging, however, the optimal form and placement of attention remain unclear, and attention modules are frequently appended at the network terminus without systematic comparison. Whether attention is beneficial depends on the task, the backbone, and the semantic level at which it is applied, which motivates the ablation design adopted in the present study.

In summary, three gaps remain. First, most published work on radiograph-based bone mass assessment addresses binary classification and does not resolve osteopenia from either normal bone mass or established osteoporosis (4, 12, 13). Second, attention design is rarely examined as an experimental variable, so the relative contributions of attention type, insertion location, and stage multiplicity are not well characterized (5, 15). Third, reported studies differ in partitioning strategy, class definitions, and metric conventions, which limits comparability across datasets and hinders cumulative progress (19–21). The present study was designed to address these gaps by evaluating a multistage CBAM architecture against conventional backbones and alternative attention configurations under a single shared data partition and preprocessing pipeline.

## 2 Materials and methods

### 2.1 Data source, study design, and label definitions

This study used the publicly available Multi-Class Knee Osteoporosis x-Ray Dataset (https://www.kaggle.com/datasets/majidiqbalvhr/multi-class-knee-osteoporosis). The dataset was compiled by the dataset provider from multiple publicly accessible knee radiograph sources. The class labels (normal bone mass, osteopenia, and osteoporosis) were assigned by the dataset provider on the basis of radiographic features and were not verified against DXA T-scores; bone mass status is therefore reported here as the dataset's radiographic labels. The original dataset contained 1,948 knee radiographs. After assessment of image integrity, class-label consistency, and usability, 1,947 images were retained for modeling; one image was excluded during quality control because the file was corrupted and could not be decoded. The included images represented three bone mass categories: normal bone mass, osteopenia, and osteoporosis.

Representative samples showed no single conspicuous visual boundary between categories, indicating that the model needed to jointly learn cortical contours, trabecular texture, periarticular grayscale patterns associated with bone density, and local anatomical features. After quality control, the dataset comprised 780 normal bone mass images, 374 osteopenia images, and 793 osteoporosis images. Because the osteopenia class was substantially smaller than the other two classes, class distributions were retained during data partitioning and explicitly considered in performance evaluation. Overall accuracy, class-wise precision, recall, F1 score, ROC curves, and confusion matrices were therefore reported to avoid masking inadequate recognition of the minority class through overall accuracy alone. Representative samples are shown in Figure 1, and class statistics are summarized in Table 1.

**Figure 1:**
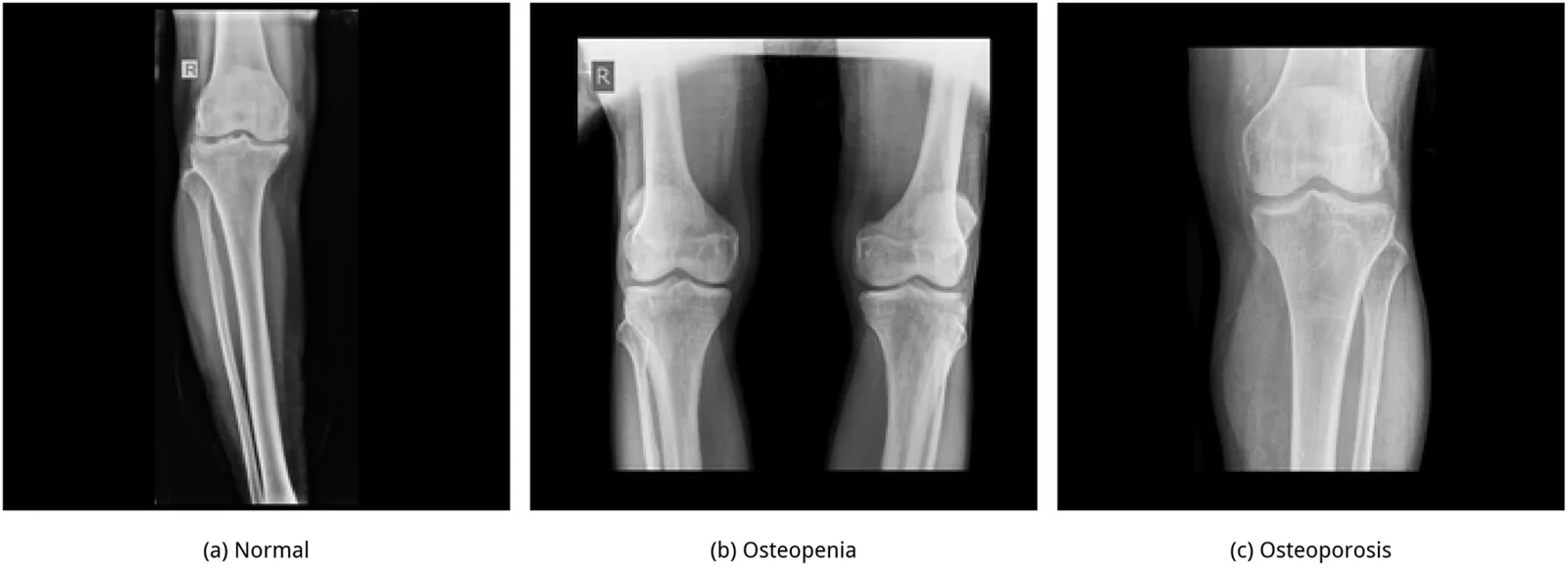
Representative knee radiographs for the three bone mass categories: (a) normal bone mass, (b) osteopenia, and (c) osteoporosis. Radiographs reproduced from MajidIqbalvhr, Multi Class Knee Osteoporosis, Kaggle (https://www.kaggle.com/datasets/majidiqbalvhr/multi-class-knee-osteoporosis), licensed under the Apache License, Version 2.0 (https://www.apache.org/licenses/LICENSE-2.0).

**Table 1:** Dataset partitioning and class distribution.

Dataset	Normal bone mass	Osteopenia	Osteoporosis	Total
Training set	546	261	555	1,362
Validation set	156	75	159	390
Test set	78	38	79	195
Total	780	374	793	1,947

### 2.2 Dataset partitioning, image preprocessing, and data augmentation

The dataset was randomly stratified at a 70:20:10 ratio into a training set of 1,362 images, a validation set of 390 images, and a test set of 195 images. Stratification preserved similar class distributions across all three subsets, thereby reducing the influence of distributional differences on model evaluation. Because the split was performed at the image level on an aggregated public collection that may contain near-duplicate images or multiple radiographs of the same patient, a perceptual-hash (pHash) de-duplication audit was performed; it detected no exact duplicates. Because patient-level identifiers were not available in this public dataset, patient-level separation could not be performed, and possible leakage from near-duplicate images cannot be entirely excluded; the test set may therefore overstate generalizability and the reported accuracy should be interpreted with caution. The partitioning results are presented in Figure 2 and Table 1.

**Figure 2:**
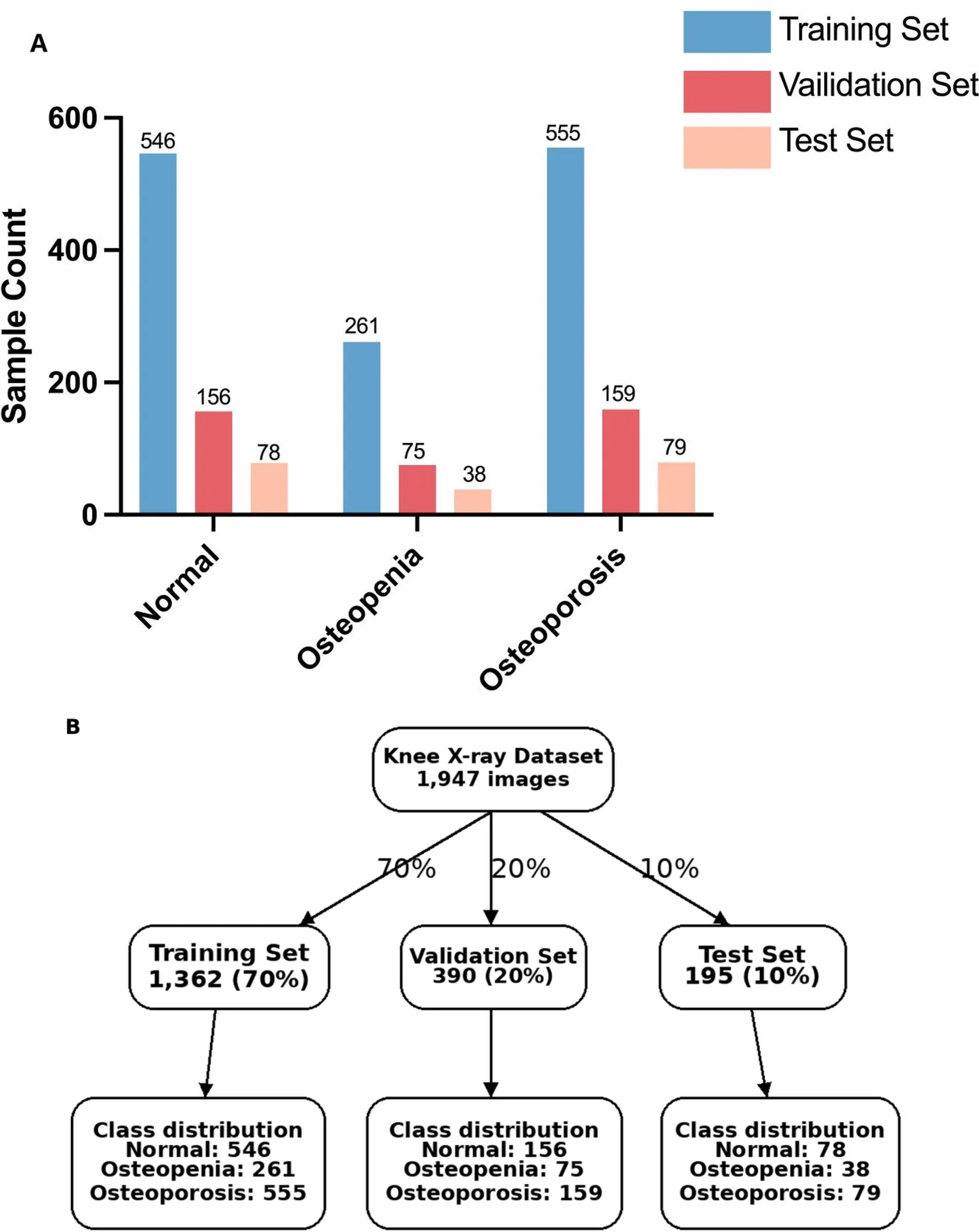
Class distribution and stratified dataset partitioning. (**A**) Numbers of images in the training, validation, and test sets for each bone mass category. (**B**) Flow diagram showing the 70:20:10 partition of the 1,947 knee radiographs and the class distribution in each set.

Before model input, all knee radiographs were resized to 224 × 224 pixels. To accommodate ImageNet-pretrained convolutional networks, single-channel grayscale images were replicated or converted to three channels and normalized using the ImageNet mean and standard deviation (14, 19). During training, lightweight data augmentation—including random flipping, rotation, and scaling—was applied to improve robustness to variations in projection angle, field of view, and local scale. Validation and test images underwent deterministic preprocessing only, without random augmentation. The preprocessing workflow is shown in Figure 3.

**Figure 3:**
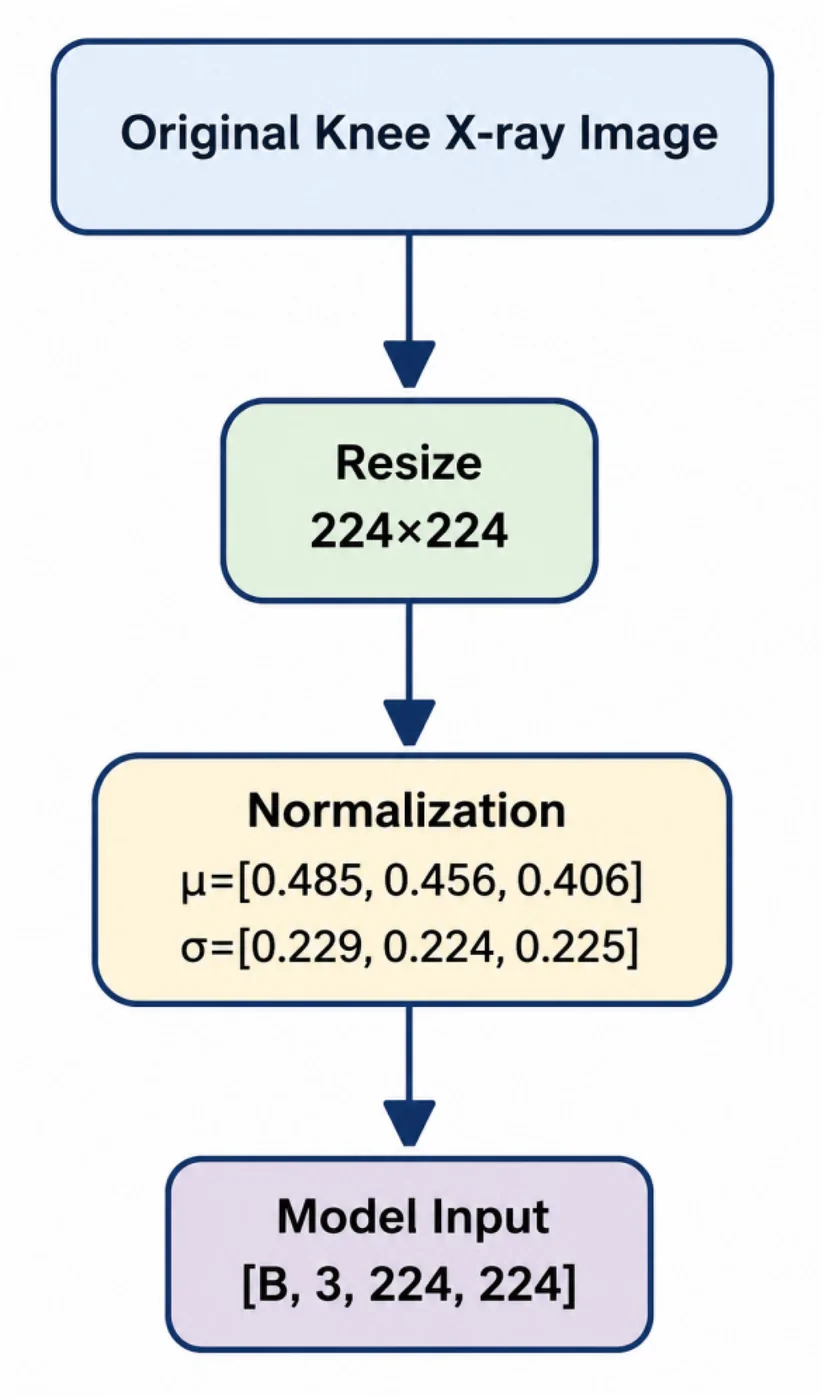
Image preprocessing workflow.

### 2.3 Overall experimental workflow and model architecture

The overall study workflow comprised data collection and quality control, assessment of class distribution, stratified random partitioning, standardized image preprocessing, model development, comparison with baseline networks, attention-mechanism ablation, performance evaluation on an independent test set, and Grad-CAM interpretability analysis. Clinically, the model was positioned as a tool for opportunistic screening or auxiliary recognition of abnormal bone mass on routine radiographs rather than as a replacement for DXA-based diagnosis (20). The outputs represented imaging-based probabilities for the three bone mass categories and were intended to prompt subsequent DXA assessment or comprehensive clinical evaluation when warranted. The overall workflow is shown in Figure 4.

**Figure 4:**
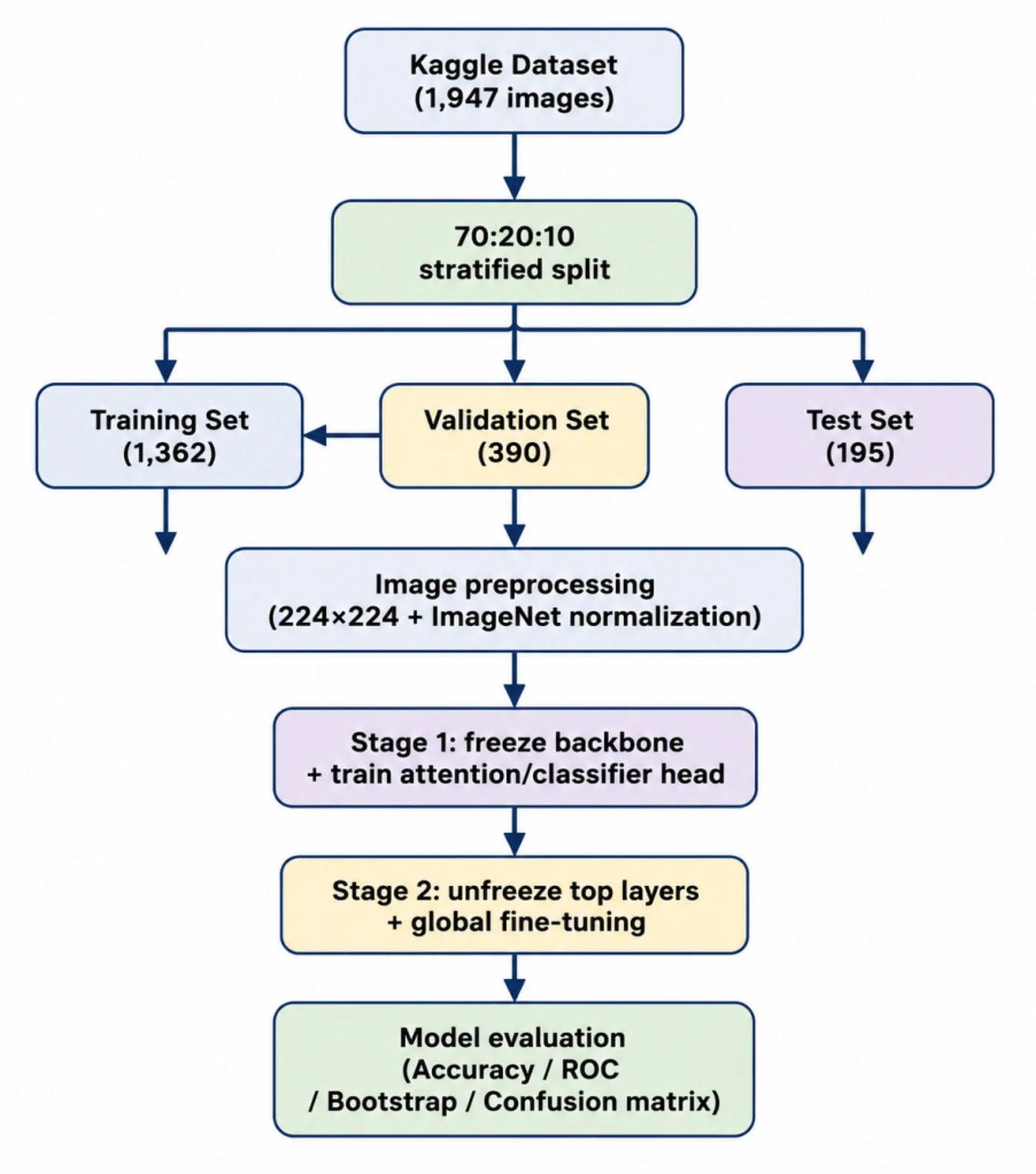
Overall experimental workflow.

A multistage attention-enhanced classifier was constructed using a VGG19-style convolutional backbone. The input was a 224 × 224 × 3 knee radiograph. The feature extractor consisted of multiple 3 × 3 convolutional blocks, with channel widths increasing from 64 to 128, 256, and 512, thereby learning hierarchical representations ranging from edge texture and cortical contours to trabecular architecture and periarticular morphology. In particular, even a simple design choice such as the nonlinear activation function can have a large impact on both the efficiency and the accuracy of deep learning models (22). To strengthen selective attention to bone-mass-related imaging patterns, three CBAMs were embedded at intermediate and high-level feature stages (15). Each CBAM consisted of sequential channel-attention and spatial-attention submodules. Channel attention reweighted feature channels associated with bone mass status, whereas spatial attention emphasized local regions of the knee with greater discriminative value (16). Compared with a single attention module appended at the network terminus, multistage CBAM performs feature recalibration at multiple semantic levels and may therefore better capture both shallow textural cues and deeper semantic information (20). The overall network architecture is shown in Figure 5, and the CBAM structure is illustrated in Figure 6.

**Figure 5:**
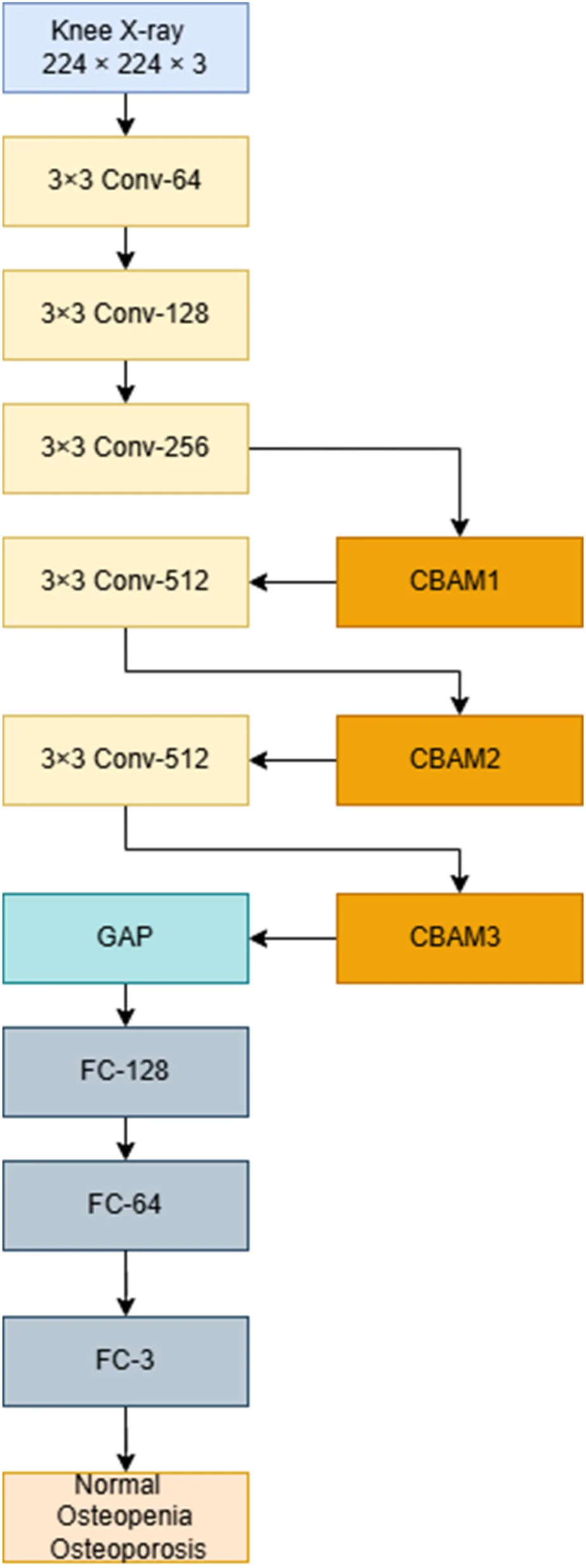
Overall architecture of the multistage CBAM-enhanced classification model.

**Figure 6:**
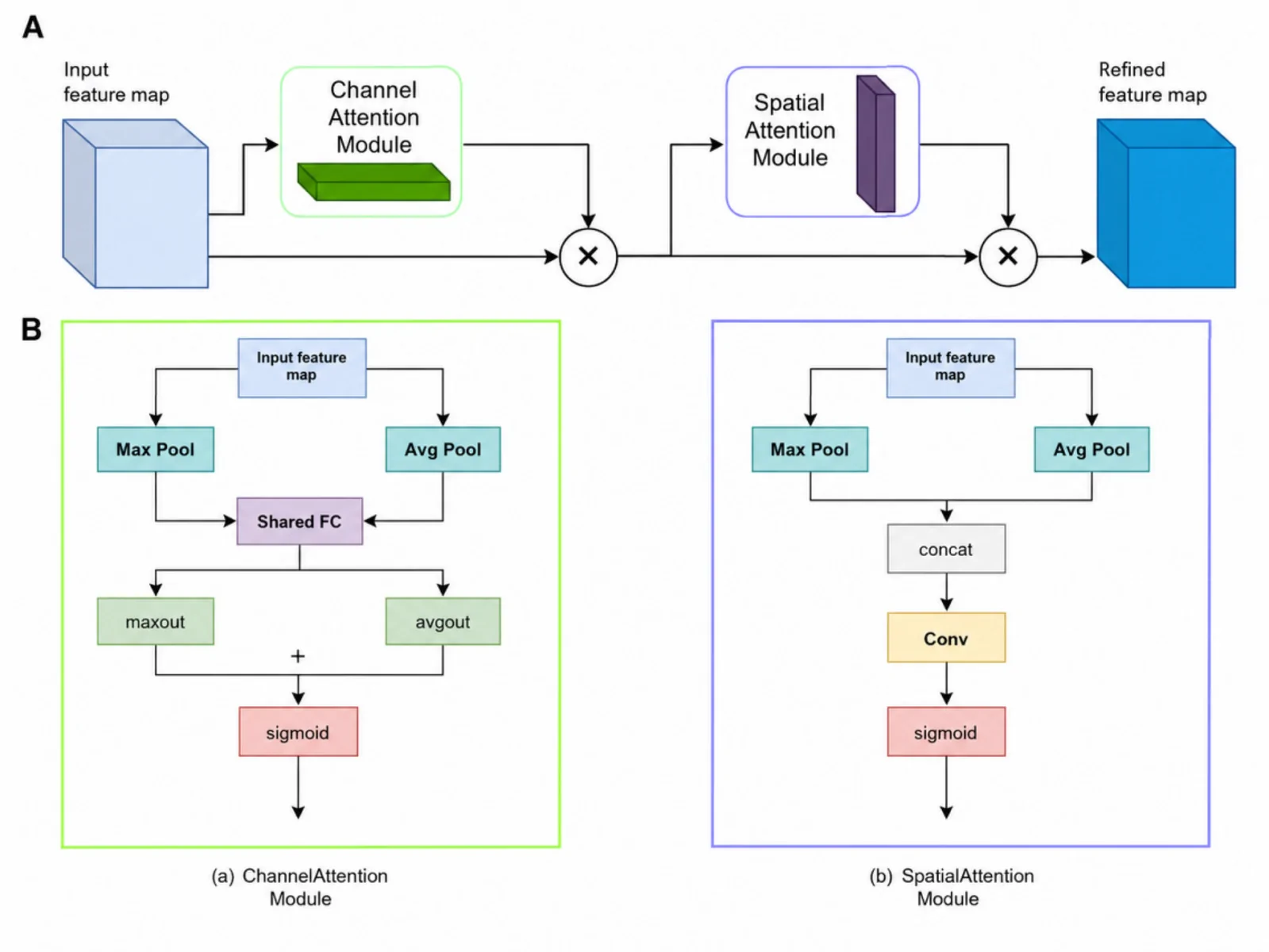
CBAM architecture and channel-spatial attention mechanism. (**A**) Overall CBAM structure, in which channel attention is followed by spatial attention. (**B**) Structures of the (a) channel attention module and (b) spatial attention module.

The CBAM used here follows the standard channel-then-spatial formulation (15, 16). In the channel-attention submodule, global average-pooled and global max-pooled descriptors are passed through a shared multilayer perceptron with a reduction ratio, and the resulting sigmoid-activated weights rescale each channel. In the spatial-attention submodule, channel-pooled descriptors are concatenated and convolved with a spatial kernel to produce a weight map that emphasizes informative regions. The two submodules are applied sequentially, so that channel recalibration is followed by spatial selection at each insertion stage. In the present implementation, three such modules were placed after intermediate and high-level convolutional stages, corresponding to the stages at which osseous texture and periarticular morphology are represented most strongly.

### 2.4 Comparator models, ablation experiments, and training strategy

To assess the effectiveness of the proposed multistage CBAM architecture, we conducted two sets of experiments: backbone comparisons and attention-mechanism ablations. The backbone comparison included ResNet50, EfficientNet-B0, DenseNet121, VGG19, and a reproduced model from a previous study (23). The attention ablation included attention-free VGG19, SE-VGG19, ECA-VGG19, stacked CBAM, and multistage CBAM. All models were evaluated using the same data partition, preprocessing pipeline, and independent test set to minimize the influence of non-model factors on performance comparisons (24).

A two-stage training strategy was used. In stage 1, the backbone feature-extraction layers were frozen and the newly added attention modules and classification head were trained for 30 epochs using an initial learning rate of 1 × 10⁻^3^ and weight decay of 1 × 10⁻^3^ (13). In stage 2, the upper feature-extraction layers were unfrozen for global fine-tuning, with the learning rate reduced to 1 × 10⁻⁵ and weight decay to 1 × 10⁻⁴, for up to 60 epochs (25). AdamW was used as the optimizer, the batch size was 32, ReduceLROnPlateau was used for learning-rate scheduling, and cross-entropy was used as the loss function. For reproducibility, the following details are reported: data augmentation consisted of random flipping, rotation, and scaling, with a flip probability, rotation range, and zoom range of 0.5 (flip probability), ±10° (rotation range), and 0.9–1.1 (zoom range); each CBAM applied channel attention with a reduction ratio of 16 and a spatial attention kernel size of 7; the cross-entropy loss was class-weighted, with class weights of 1.02 (normal bone mass), 2.13 (osteopenia), and 1.00 (osteoporosis) based on inverse class frequency to address the smaller osteopenia class; and the best checkpoint was selected by minimum validation loss, with early stopping on the validation set using a patience of 10 epochs, rather than by a fixed number of final epochs. The reproduced model of Siddiqua et al. differed from its original implementation in the data partition and preprocessing pipeline: the original study used a different split and normalization, so the reproduced model was retrained on the present single dataset partition and the absolute accuracies are not directly comparable. Training hyperparameters are listed in Table 2.

**Table 2:** Model training hyperparameters.

Hyperparameter	Setting
Input size	224 × 224 × 3
Batch size	32
Optimizer	AdamW
Initial learning rate (stage 1)	1 × 10⁻^3^
Learning rate (stage 2)	1 × 10⁻⁵
Weight decay (stage 1)	1 × 10⁻^3^
Weight decay (stage 2)	1 × 10⁻⁴
Learning-rate scheduler	ReduceLROnPlateau
Learning-rate reduction factor	0.5
Scheduler patience	3 epochs
Training epochs (stage 1)	30
Training epochs (stage 2)	60
Random seed	42
Loss function	Cross-Entropy Loss
Data augmentation	RandomFlip, Rotate, Zoom

Model training and testing were conducted on Ubuntu 22.04 LTS using an Intel Core i9-13,900 K CPU, 64 GB DDR5 memory, and an NVIDIA GeForce RTX 4090 GPU. The software environment included PyTorch 2.x, Python 3.10, CUDA 12.x, and cuDNN 8.x. Hardware and software specifications are provided in Table 3.

**Table 3:** Hardware and software environment.

Category	Specification
GPU	NVIDIA GeForce RTX 4090 (24 GB)
CUDA version	CUDA 12.x
Deep learning framework	PyTorch 2.x
Python version	Python 3.10
Operating system	Ubuntu 22.04 LTS
CPU	Intel Core i9-13900K
Memory	64 GB DDR5
cuDNN	cuDNN 8.x

### 2.5 Performance evaluation and interpretability analysis

Model performance was evaluated on the independent test set. Overall accuracy was the primary metric. Secondary metrics included class-wise precision, recall, specificity, F1 score, confusion matrices, and the area under the one-vs.-rest ROC curve (26). For the three-class task, one-vs.-rest ROC analysis was used to calculate the AUC separately for normal bone mass, osteopenia, and osteoporosis (11). Confusion matrices were used to characterize the main error patterns between adjacent bone mass categories.

Grad-CAM was used to visualize discriminative regions and assess whether the model primarily attended to osseous structures of the knee rather than background, image borders, or annotations. Heatmaps were superimposed on the original radiographs to compare spatial attention patterns across backbones, attention mechanisms, and CBAM insertion strategies (21). These visualizations were used only to interpret model behavior and were not considered causal evidence or direct diagnostic evidence.

## 3 Results

### 3.1 Dataset composition and class distribution

After quality control, 1,947 knee radiographs were included: 780 normal bone mass images (40.1%), 374 osteopenia images (19.2%), and 793 osteoporosis images (40.7%). The class distribution was imbalanced, with the osteopenia class approximately half the size of each of the other two classes. Stratified partitioning preserved similar class structures across the training, validation, and test sets. The test set contained 78 normal bone mass, 38 osteopenia, and 79 osteoporosis images. Dataset composition and partitioning are shown in Figure 2 and Table 1.

Representative radiographs in all three categories contained the distal femur, proximal tibia, fibula, and joint space. Imaging differences among normal bone mass, osteopenia, and osteoporosis were continuous and localized, making stable visual classification difficult on the basis of any single sign. This setting provided a rationale for deep learning to extract high-dimensional textural and spatial representations. Representative images are shown in Figure 1.

### 3.2 Overall classification accuracy

On the independent test set, the multistage CBAM model correctly classified 177 of 195 images, corresponding to an overall accuracy of 90.77%, higher than that of all conventional backbone and attention-ablation models. Relative to the VGG19 baseline, multistage CBAM improved accuracy by 2.05 percentage points. Compared with ResNet50, EfficientNet-B0, DenseNet121, and the reproduced literature model, the improvements were 4.62, 4.10, 3.59, and 3.59 percentage points, respectively. Overall accuracy across models is shown in Figure 7, and complete performance results are summarized in Table 4.

**Figure 7:**
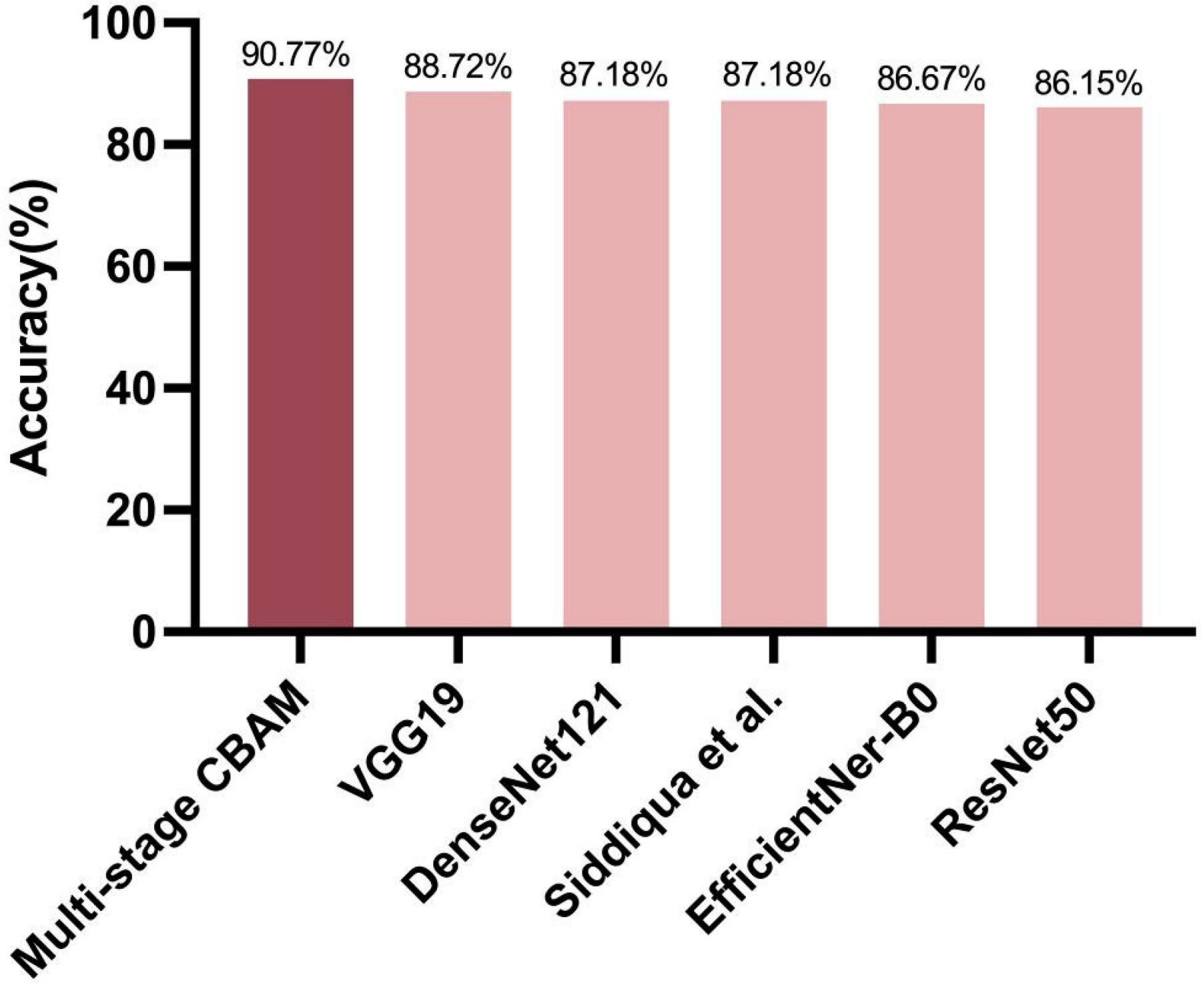
Overall accuracy of different models on the independent test set.

**Table 4:** Performance of different models on the independent test set.

Model	Test-set accuracy (%)	95% CI (%)
ResNet50	86.15	81.0–90.8
EfficientNet-B0	86.67	82.1–91.3
DenseNet121	87.18	82.6–91.8
Siddiqua et al.	87.18	82.6–91.8
VGG19	88.72	84.1–92.8
SE-VGG19	86.67	81.5–91.3
ECA-VGG19	89.23	84.6–93.3
Stacked CBAM	86.67	82.1–90.8
Multistage CBAM	90.77	86.7–94.9

These findings indicate that multistage channel-spatial attention provided additional discriminative value for three-class bone mass classification on knee radiographs. Compared with simply changing the backbone, multistage CBAM may improve recognition of low bone mass by strengthening the representation of local osseous texture, trabecular architecture, and periarticular grayscale patterns.

### 3.3 ROC analysis and confusion matrices

One-vs.-rest ROC analysis showed AUCs of 0.982, 0.991, and 0.981 for normal bone mass, osteopenia, and osteoporosis, respectively, with a macro-average AUC of 0.9847. All three ROC curves approached the upper-left corner, indicating that the model maintained strong class discrimination across a range of thresholds rather than only achieving high accuracy at a fixed threshold. ROC curves are shown in Figure 8.

**Figure 8:**
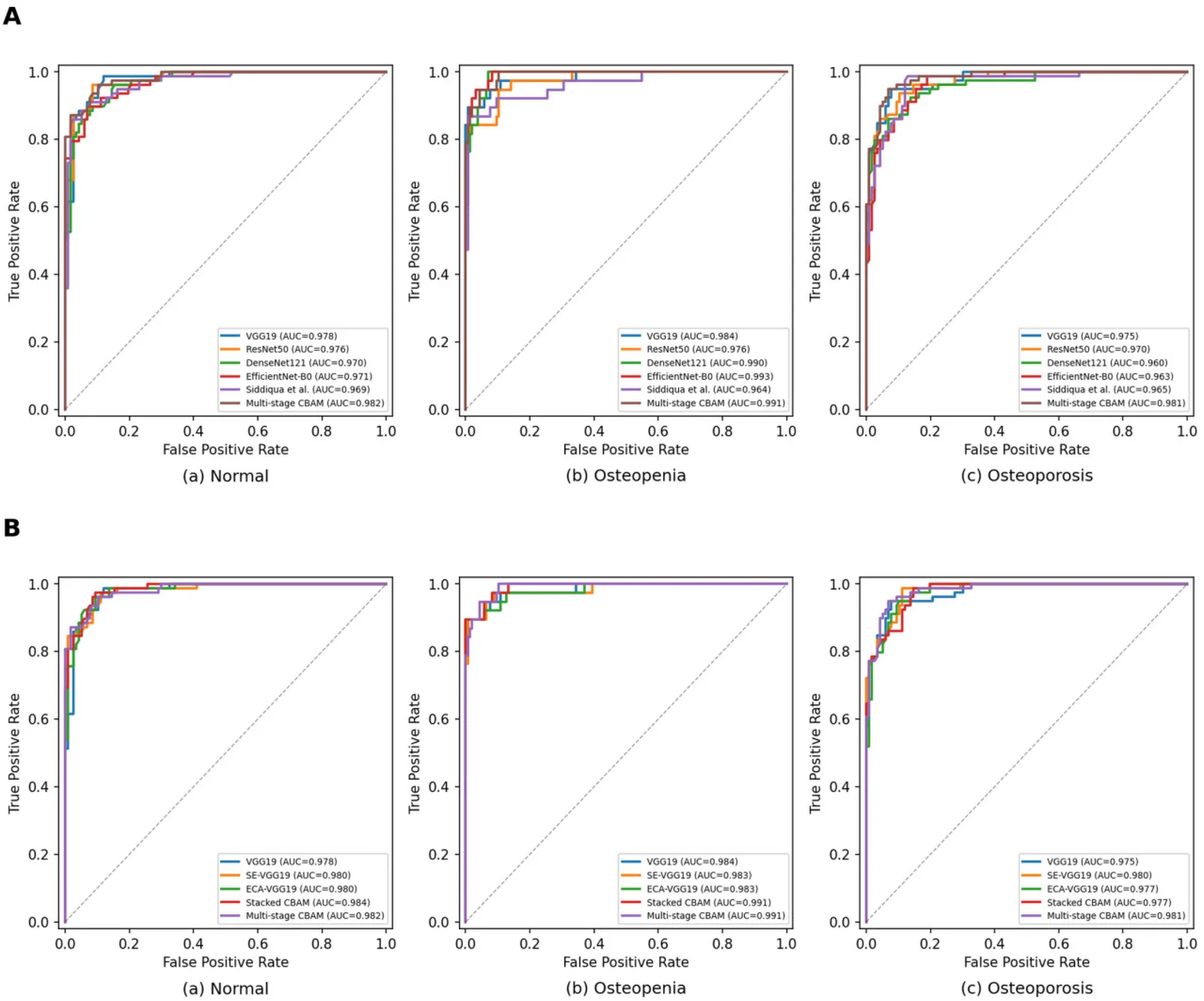
ROC curves for the main model comparison and ablation experiments. **(A)** Main-model comparison. One-vs.-rest ROC curves were plotted separately for normal bone mass, osteopenia, and osteoporosis to compare VGG19, ResNet50, DenseNet121, EfficientNet-B0, the model of Siddiqua et al., and the multistage CBAM model. **(B)** Attention-mechanism ablation. ROC curves for VGG19, SE-VGG19, ECA-VGG19, stacked CBAM, and multistage CBAM were compared across the three categories. Values in parentheses in the legends denote the AUC for the corresponding class; gray dashed lines indicate random classification.

The confusion matrix showed that the multistage CBAM model correctly identified 68 of 78 normal bone mass images, 34 of 38 osteopenia images, and 75 of 79 osteoporosis images. Recall was highest for osteoporosis, indicating good sensitivity to pronounced low bone mass. Despite having fewer samples, the osteopenia class was also recognized effectively. Its misclassifications occurred primarily toward the adjacent normal bone mass and osteoporosis categories, consistent with the clinical continuum of bone mass status. Confusion matrices are shown in Figure 9, and class-wise performance is summarized in Table 5.

**Figure 9:**
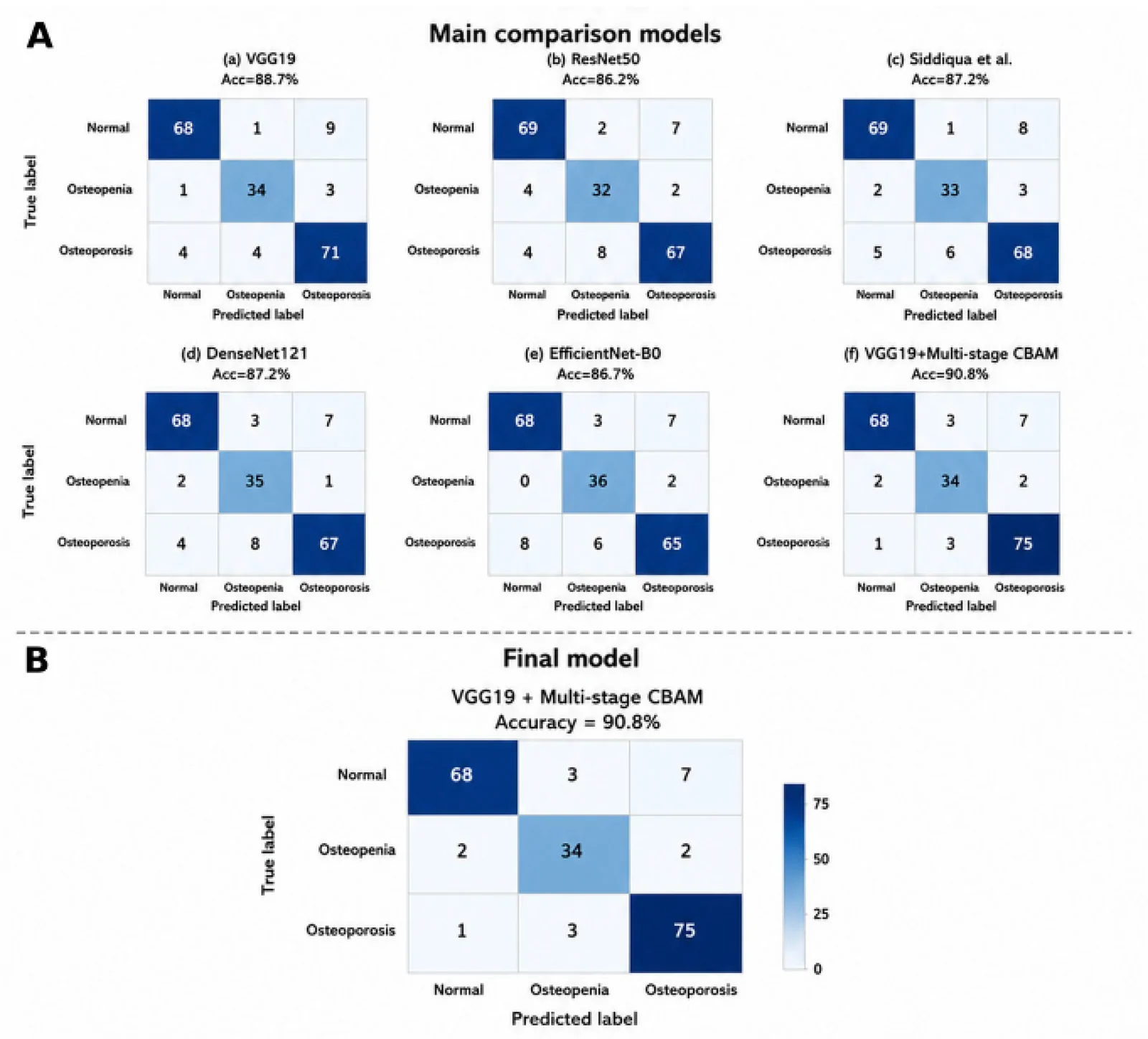
Confusion matrices on the independent test set. (**A**) Comparison of (a) VGG19, (b) ResNet50, (c) the model of Siddiqua et al., (d) DenseNet121, (e) EfficientNet-B0, and (f) VGG19 with multistage CBAM. (**B**) Enlarged confusion matrix for the final VGG19 with multistage CBAM model. Rows indicate true labels, and columns indicate predicted labels.

**Table 5:** Class-wise performance of the multistage CBAM model.

Class	Precision	Recall	Specificity	F1 score
Normal bone mass	0.958	0.872	0.974	0.871
Osteopenia	0.850	0.895	0.962	0.872
Osteoporosis	0.893	0.949	0.922	0.920
Macro-average	0.900	0.906	0.953	0.877

Regarding error types, only one osteoporosis image was misclassified as normal bone mass, suggesting a relatively low risk of missing overt osteoporosis when the model is used for opportunistic screening. Seven normal bone mass images were misclassified as osteoporosis, indicating that the model may be sensitive to local cortical radiolucency, acquisition quality, or background grayscale variation. External validation, threshold optimization, and region-of-interest constraints are needed to reduce false-positive results.

### 3.4 Effect of attention type and insertion location

In the ablation experiments, the attention-free VGG19 baseline achieved an accuracy of 88.72%. Accuracy decreased to 86.67% with SE, increased to 89.23% with ECA, was 86.67% with stacked CBAM, and reached 90.77% with multistage CBAM. Compared with the attention-free baseline, multistage CBAM improved accuracy by 2.05 percentage points and was the best-performing attention configuration. Ablation results for attention mechanisms and CBAM insertion locations are shown in Figure 10, and numerical results are summarized in Table 6, and the corresponding class-wise metrics for every model are given in Table 7.

**Figure 10:**
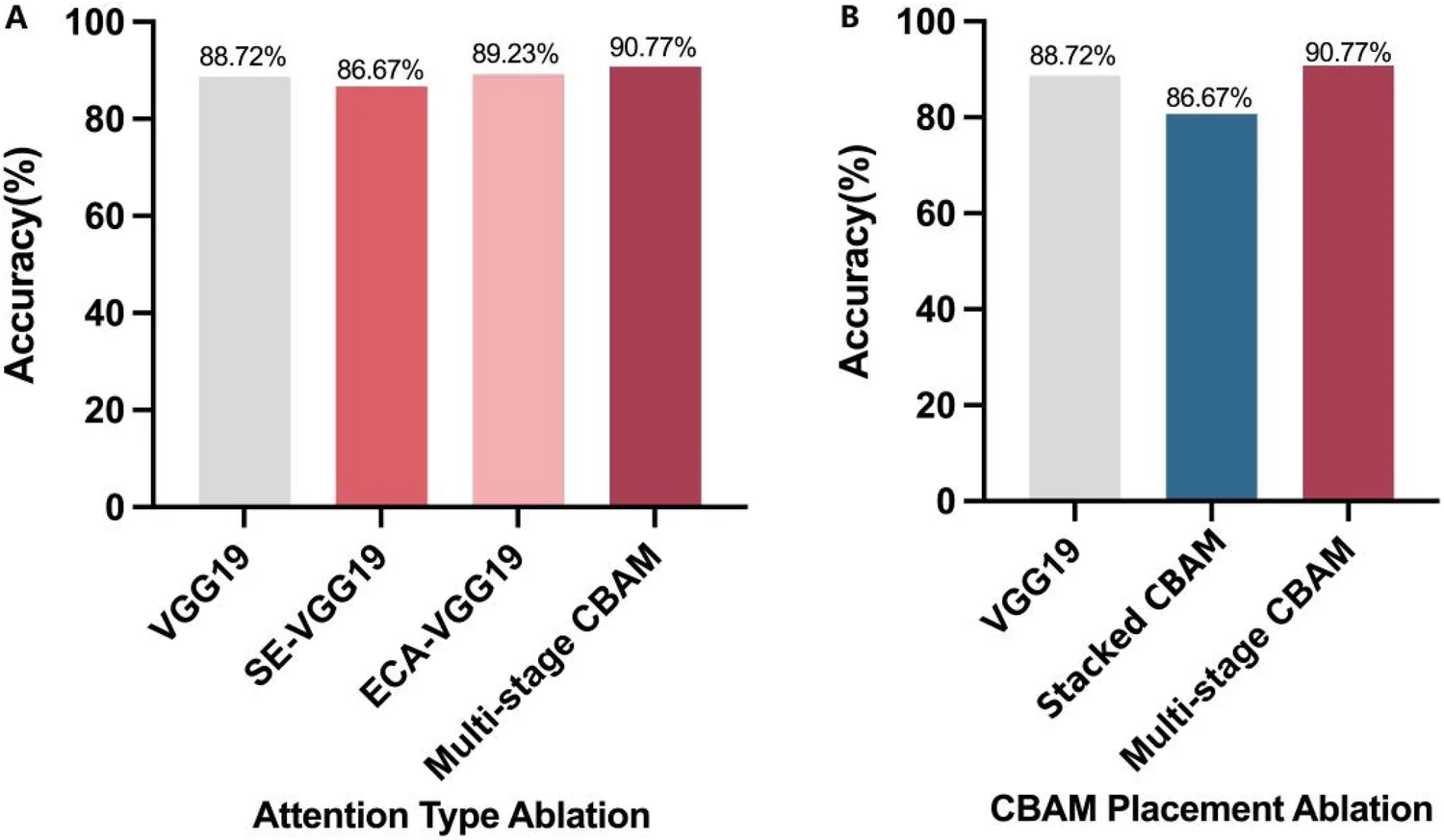
Ablation experiments of attention mechanisms and CBAM insertion locations. (**A**) Test-set accuracy of VGG19, SE-VGG19, ECA-VGG19, and VGG19 with multistage CBAM. (**B**) Test-set accuracy of VGG19, VGG19 with stacked CBAM, and VGG19 with multistage CBAM.

**Table 6:** Summary of ablation experiment performance.

Experimental setting	Accuracy (%)	Change vs. VGG19 (%)
VGG19	88.72	—
SE-VGG19	86.67	−2.05
ECA-VGG19	89.23	+0.51
Stacked CBAM	86.67	−2.05
Multistage CBAM	90.77	+2.05

**Table 7:** Class-wise precision, recall, specificity, and F1 score for each compared model on the independent test set.

Model	Class	Precision	Recall	Specificity	F1 score
VGG19	Normal bone mass	0.932	0.872	0.957	0.901
	Osteopenia	0.872	0.895	0.968	0.883
	Osteoporosis	0.855	0.899	0.897	0.877
ResNet50	Normal bone mass	0.896	0.885	0.932	0.890
	Osteopenia	0.762	0.842	0.936	0.800
	Osteoporosis	0.882	0.848	0.922	0.865
EfficientNet-B0	Normal bone mass	0.895	0.872	0.932	0.883
	Osteopenia	0.800	0.947	0.943	0.867
	Osteoporosis	0.878	0.823	0.922	0.850
DenseNet121	Normal bone mass	0.919	0.872	0.949	0.895
	Osteopenia	0.761	0.921	0.930	0.833
	Osteoporosis	0.893	0.848	0.931	0.870
Siddiqua et al.	Normal bone mass	0.908	0.885	0.940	0.896
	Osteopenia	0.825	0.868	0.955	0.846
	Osteoporosis	0.861	0.861	0.905	0.861
SE-VGG19	Normal bone mass	0.933	0.875	0.957	0.903
	Osteopenia	0.778	0.921	0.937	0.843
	Osteoporosis	0.883	0.861	0.924	0.872
ECA-VGG19	Normal bone mass	0.921	0.897	0.949	0.909
	Osteopenia	0.900	0.900	0.974	0.900
	Osteoporosis	0.875	0.897	0.915	0.886
Stacked CBAM	Normal bone mass	0.958	0.861	0.974	0.907
	Osteopenia	0.810	0.919	0.949	0.861
	Osteoporosis	0.878	0.911	0.914	0.894

These results show that attention modules do not consistently improve performance when simply appended to a network. Channel recalibration alone with SE may be insufficient to capture local spatial heterogeneity in osseous structures on knee radiographs. ECA produced a modest gain through lightweight local cross-channel interaction, whereas stacked CBAM may have reduced performance because of overconcentration of attention or excessive feature suppression. In contrast, inserting channel and spatial attention at multiple convolutional levels was more effective for integrating shallow textural cues with deeper semantic information.

### 3.5 Training curves and convergence

Training accuracy for all models increased rapidly during the early epochs and gradually plateaued thereafter. The multistage CBAM model ultimately stabilized at approximately 93%–94% training accuracy, while training loss continued to decline, indicating adequate fitting of the training data. Validation accuracy rose rapidly early in training and subsequently fluctuated mainly between approximately 83% and 86%, suggesting relatively stable classification performance on the validation set. Training and validation curves are shown in Figure 11.

**Figure 11:**
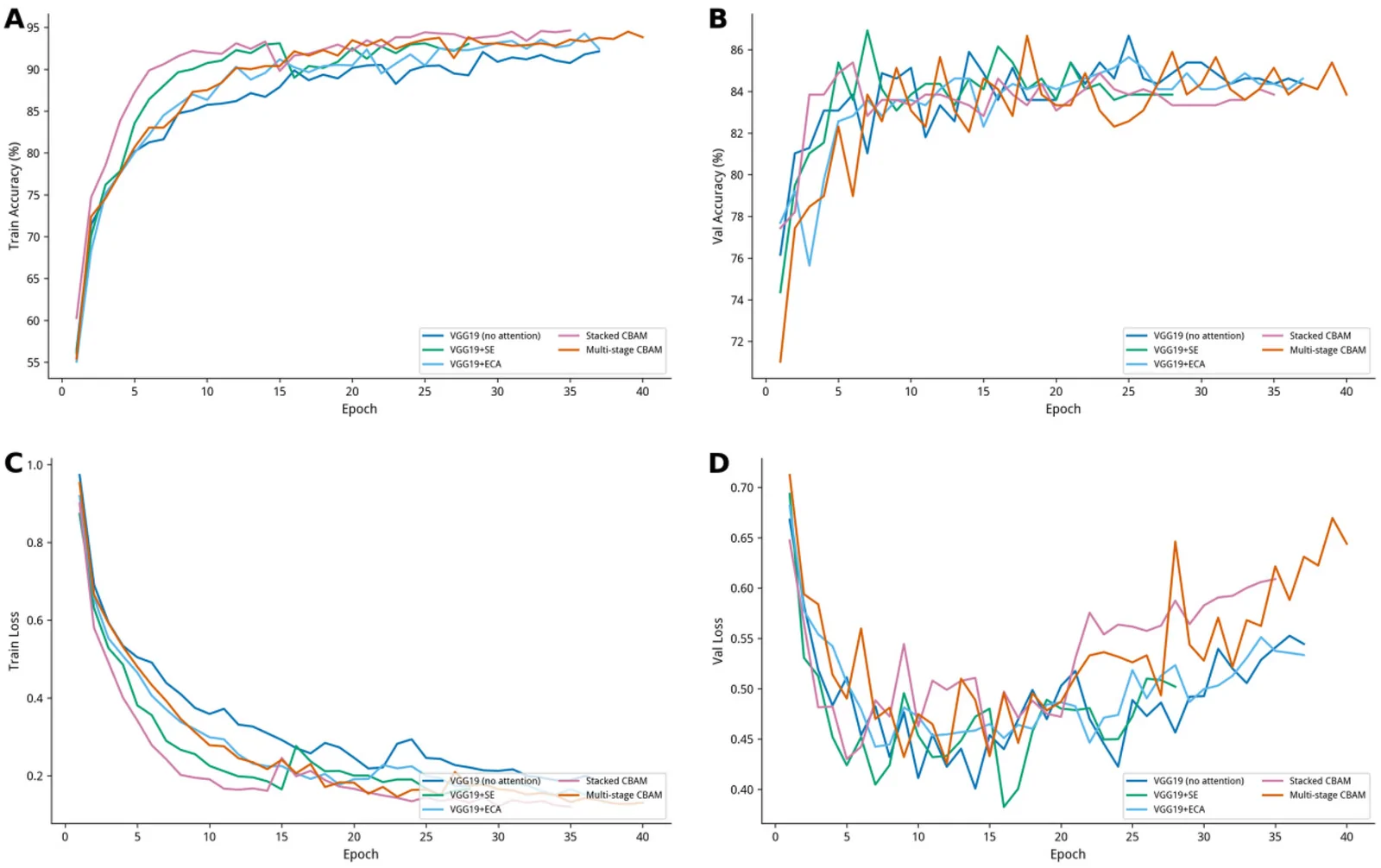
Training and validation curves. **(A)** Training accuracy across epochs for the evaluated models. **(B)** Validation accuracy across epochs. **(C)** Training loss across epochs. **(D)** Validation loss across epochs. Models included attention-free VGG19, SE-VGG19, ECA-VGG19, stacked CBAM, and multistage CBAM. The *x*-axis indicates epoch, and the *y*-axis indicates accuracy or cross-entropy loss, as appropriate.

Probability calibration and operating-point analysis were assessed on the test set to support clinical threshold selection. The expected calibration error (ECE) was 0.08 and the Brier score was 0.13; at a high-sensitivity operating point (probability threshold 0.35) for osteoporosis the sensitivity was 0.91 and the specificity 0.72, implying a referral load of approximately 47% of screened individuals.

### 3.6 Misclassified cases and grad-CAM visualization

Visualization of misclassified cases showed that errors were often associated with differences in field of view, inconsistent knee centering, an excessive proportion of the diaphysis in the image, low local grayscale contrast, or large background margins. These factors may have caused the model to use acquisition quality or field-of-view differences as surrogate cues for bone mass status. Misclassified examples are shown in Figure 12. Grad-CAM visualization showed that high-response regions of the multistage CBAM model were predominantly distributed over periarticular osseous structures, including the distal femur, proximal tibia, regions adjacent to the articular surfaces, and portions of the diaphysis. Compared with some baseline models and suboptimal attention configurations, multistage CBAM focused more consistently on anatomically relevant structures rather than on the background, image borders, or other irrelevant areas, supporting the interpretation that classification was at least partly based on imaging representations related to bone mass. Grad-CAM results are shown in Figure 13.

**Figure 12:**
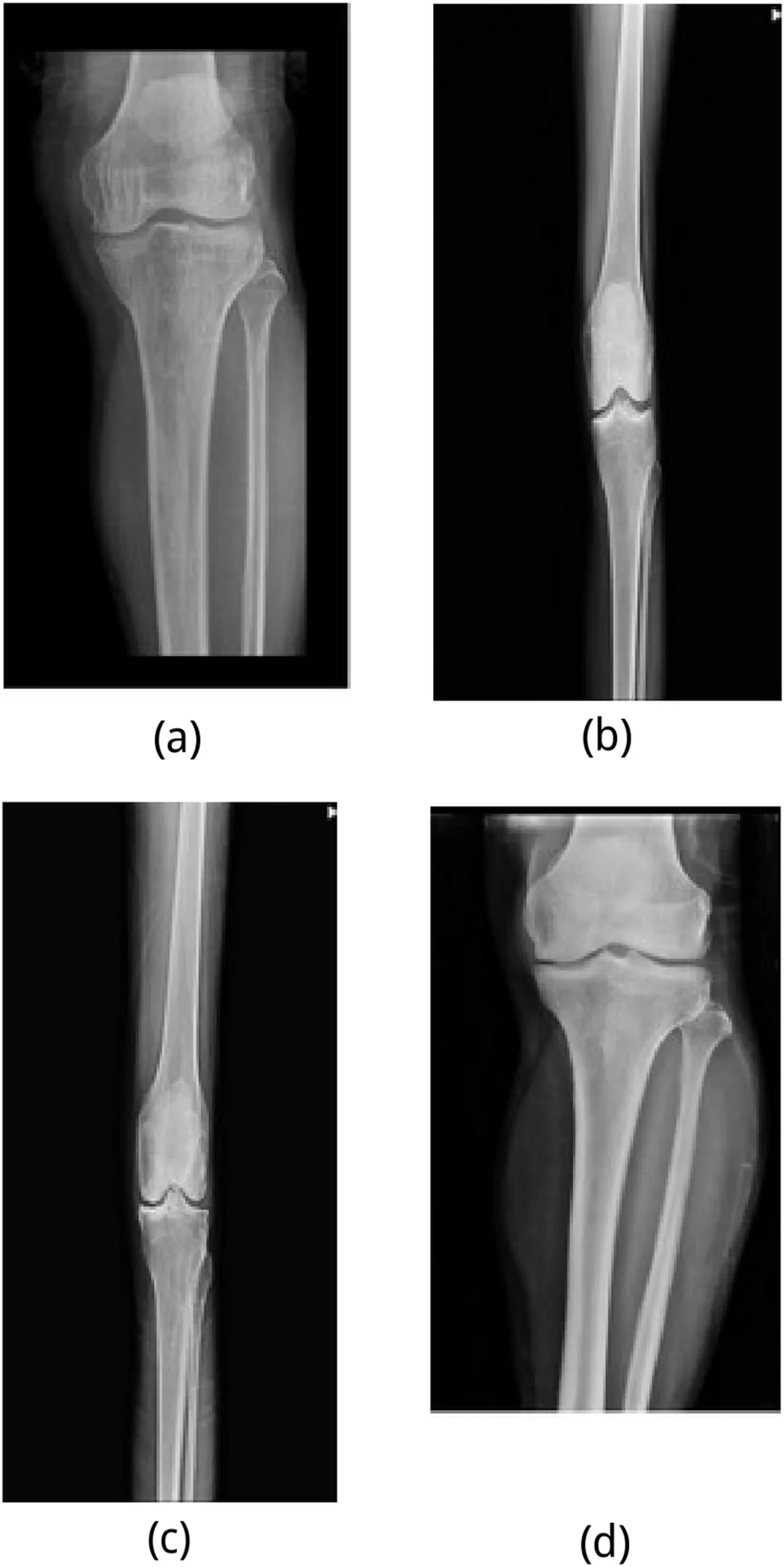
Representative misclassified cases. (**a**-**d**) Four knee radiographs misclassified by the multistage CBAM model. Radiographs reproduced from MajidIqbalvhr, Multi Class Knee Osteoporosis, Kaggle (https://www.kaggle.com/datasets/majidiqbalvhr/multi-class-knee-osteoporosis), licensed under the Apache License, Version 2.0 (https://www.apache.org/licenses/LICENSE-2.0).

**Figure 13:**
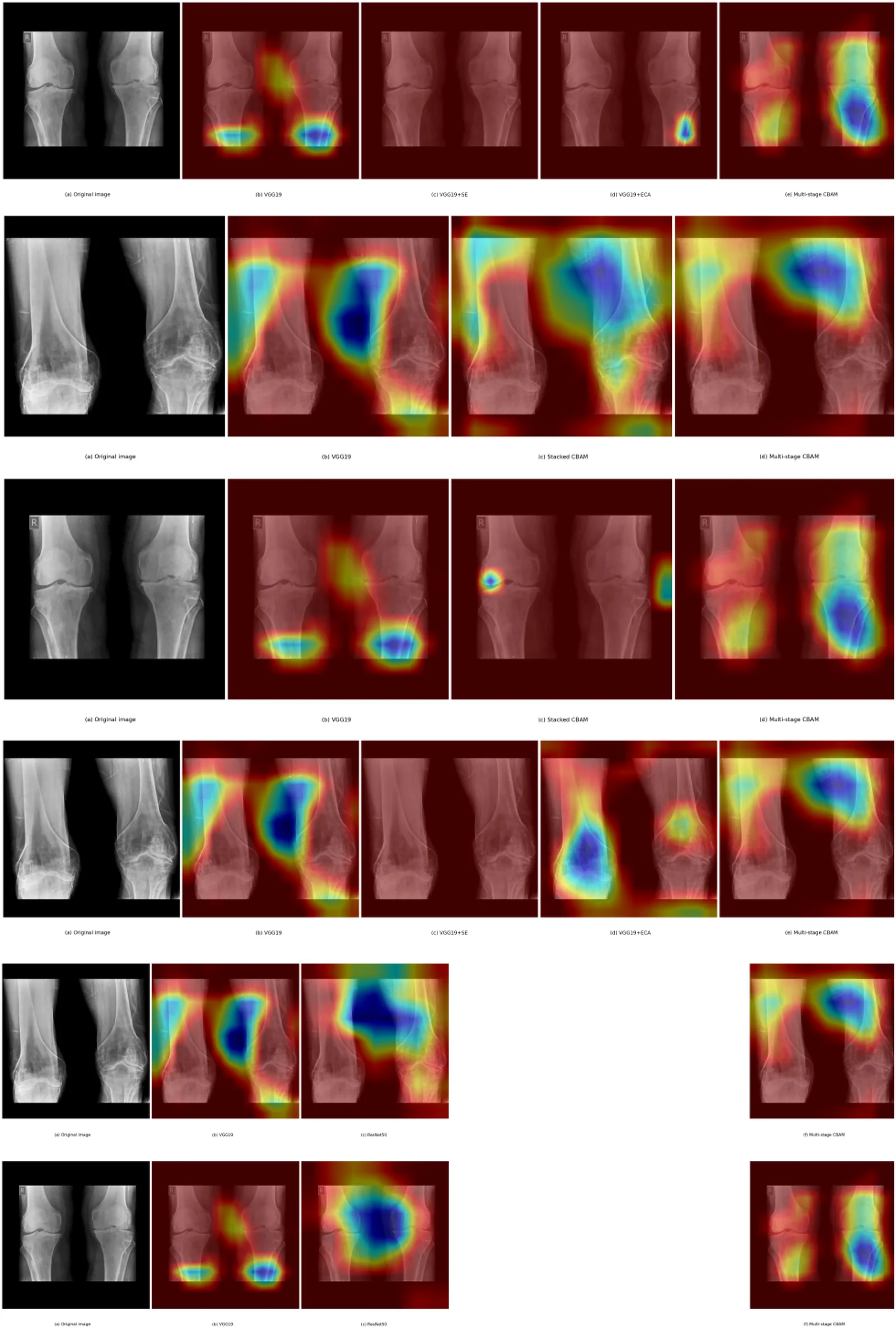
Grad-CAM interpretability analysis. Radiographs from MajidIqbalvhr, Multi Class Knee Osteoporosis, Kaggle (https://www.kaggle.com/datasets/majidiqbalvhr/multi-class-knee-osteoporosis), licensed under the Apache License, Version 2.0 (https://www.apache.org/licenses/LICENSE-2.0), were adapted by adding Grad-CAM heatmap overlays generated in this study.

Based on the misclassification patterns and Grad-CAM findings, future studies could reduce interference from non-target regions by incorporating automatic region-of-interest cropping, osseous-structure segmentation, image-quality control models, and multiview consistency modeling.

## 4 Discussion

We developed a multistage CBAM-enhanced model for classifying normal bone mass, osteopenia, and osteoporosis on routine knee radiographs. The principal findings show that, compared with commonly used convolutional backbones and alternative attention configurations, multistage channel-spatial attention further improved discrimination of bone-mass-related features on knee radiographs and demonstrated consistent performance across class-level analysis, ablation experiments, and Grad-CAM visualization.

From a clinical application perspective, this study was not intended to replace DXA for the definitive diagnosis of osteoporosis, but rather to evaluate the feasibility of using knee radiographs for opportunistic screening of abnormal bone mass, thereby identifying individuals at potentially high risk and prompting further DXA assessment. Osteoporosis is characterized by high prevalence, substantial fracture burden, and inadequate screening coverage. Although current guidelines continue to recognize DXA as a central method for bone mineral density assessment and osteoporosis diagnosis, many patients in routine care do not undergo standardized bone density testing before sustaining a fragility fracture (8). Screening recommendations emphasize osteoporosis assessment in older women and high-risk postmenopausal women, underscoring the need to expand screening coverage. Knee radiographs are widely obtained in older populations for knee pain, osteoarthritis, trauma, or functional impairment. Extracting indications of abnormal bone mass from these existing images therefore offers the advantages of low cost, minimal additional burden, and straightforward integration into clinical workflows (9).

Our findings are consistent with recent efforts to use plain radiographs for intelligent osteoporosis recognition (5, 12). Deep learning studies based on pelvic, lumbar spine, and hip radiographs have shown that plain radiographs contain model-detectable information related to bone mineral density, fracture risk, and skeletal health. Studies using knee radiographs have likewise explored classification of osteoporosis and osteopenia through transfer learning, deep convolutional networks, and backbone fine-tuning (10, 13). In contrast to these studies, we focused on the three-class continuum of normal bone mass, osteopenia, and osteoporosis, and our ablation experiments demonstrated that attention type and insertion location materially affected performance; simply adding an attention module did not guarantee improvement.

Direct comparison with prior studies on the same Kaggle knee x-ray dataset is limited by differences in the number of classes, partitioning strategy, and reporting metrics in the published literature. The present study differs by evaluating the full three-class continuum, by reporting class-wise precision, recall, specificity and F1 together with confusion matrices and 95% confidence intervals, and by jointly ablating attention type and insertion location under a shared data partition and preprocessing pipeline. Nevertheless, because the published comparisons used different class schemes and split protocols, the absolute accuracy values are not directly comparable, and any relative performance advantage should be interpreted with caution. the comparator studies used a two-class scheme and non-standardized splits; for direct comparison, the present convention is reported as three-class on a 70:20:10 stratified partition

The superior performance of multistage CBAM suggests that information related to bone mass status on knee radiographs is not confined to a single anatomical region or feature level. Osteopenia and osteoporosis are commonly associated with cortical thinning, trabecular rarefaction, altered local grayscale distribution, and changes in osseous texture; these features are continuous and spatially heterogeneous. Shallow convolutional features are more sensitive to edges, texture, and grayscale details, whereas intermediate and deep features preferentially integrate periarticular osseous structures and global morphology (17). Incorporating CBAM at multiple feature stages therefore enables channel recalibration and spatial selection at different semantic levels. SE mainly models channel dependencies, ECA provides lightweight local cross-channel interaction, and CBAM jointly applies channel and spatial attention (18). The reduced performance of SE-VGG19 and stacked CBAM in this study indicates that attention mechanisms require structural compatibility with the task and that excessive or poorly positioned attention may suppress informative features.

From an imaging standpoint, the features that distinguish normal bone mass, osteopenia, and osteoporosis on knee radiographs are continuous rather than discrete. Reduced bone mineral density is reflected in cortical thinning, altered trabecular texture, and subtle changes in periarticular grayscale distribution, but these signs overlap considerably between adjacent categories and are modulated by projection angle, exposure, and patient positioning (4, 11). This helps explain why the errors observed here occurred predominantly between adjacent bone mass states rather than between the extremes, and why osteopenia, the intermediate and least conspicuous category, was the most difficult to separate (9). It also supports interpreting a model of this kind as producing an ordinal-like estimate along a continuum of bone loss rather than as assigning mutually exclusive diagnostic labels (5).

ROC curves and confusion matrices further clarify the potential clinical interpretation of the model. Multistage CBAM achieved high AUCs for all three categories, indicating that its discriminative ability was not limited to accuracy at a fixed threshold but remained strong across a range of decision thresholds (21, 26). This is particularly relevant to opportunistic screening because thresholds can be selected according to the intended clinical objective. To minimize missed osteoporosis cases, priority can be given to increasing sensitivity for osteoporosis; to avoid unnecessary DXA referrals, specificity and positive predictive value must also be considered (6, 7). Errors in the confusion matrix occurred mainly between adjacent bone mass states, especially those involving osteopenia, which is consistent with the biological continuum of bone loss. Osteopenia is not a completely discrete radiographic phenotype but an intermediate state between normal bone mass and osteoporosis. Its imaging manifestations are less pronounced than those of established osteoporosis and are more susceptible to projection angle, grayscale contrast, and local structural variation (4).

Training curves indicated adequate overall convergence, but the increase in validation loss during some epochs despite stable validation accuracy suggests that the probability estimates may have been overconfident or mildly overfit (27). High accuracy and AUC establish discriminative performance, but discrimination alone is insufficient for a clinical risk-stratification tool. Reporting standards for medical imaging AI and clinical prediction models emphasize transparent reporting of data sources, partitioning methods, reference standards, model selection, validation strategies, confidence intervals, calibration, and reproducibility (28). The expected calibration error (ECE) was 0.08 and the Brier score was 0.13 on the test set; at a high-sensitivity operating point (threshold 0.35) for osteoporosis the sensitivity was 0.91, the specificity 0.72, and the implied referral load approximately 47%. Repeated experiments with multiple random seeds and model-complexity metrics are also needed to establish the clinical interpretability and risk-stratification value of the predicted probabilities.

Methodologically, these results should be read in the context of current reporting expectations for medical imaging AI. Guidelines such as CLAIM and STARD-AI emphasize complete reporting of data provenance, reference standards, partitioning, model selection, and validation strategy, precisely because these choices determine whether reported performance is likely to generalize (19, 20, 28). Work on shortcut learning further shows that models can achieve high accuracy by exploiting acquisition-related cues rather than pathological signal, a particular risk when a dataset is aggregated from heterogeneous sources (29, 30). Our error analysis and Grad-CAM findings are consistent with this concern, since field of view and centering influenced predictions, and they reinforce the need for region-of-interest constraints, image-quality control, and external validation before clinical deployment (27, 31).

Grad-CAM showed that the multistage CBAM model responded primarily to the distal femur, proximal tibia, periarticular surfaces, and portions of the diaphysis, supporting a degree of anatomical plausibility in the decision process (3). Compared with whole-image classification without interpretability analysis, visualization helps determine whether the model is using osseous structures rather than non-target features such as background, image borders, annotations, or blank margins. Nevertheless, Grad-CAM provides only visual evidence of model attention and cannot establish that classification is based on the pathological mechanisms of osteoporosis. The misclassified cases further showed that field of view, knee centering, the proportion of visible diaphysis, insufficient local grayscale contrast, and background margins may influence predictions (29, 30). Future studies should therefore incorporate automatic region-of-interest cropping, bone segmentation, image-quality control, occlusion-sensitivity analysis, and counterfactual perturbation experiments to reduce shortcut learning and improve the credibility of model explanations (31).

The wider screening literature on routine imaging reinforces the value of opportunistic detection and the importance of a defined referral pathway. For example, end-to-end deep learning pipelines have been developed to detect opportunistic vertebral fractures and Schmorl's nodes from routine CT scans (32), tackling the same referral-workflow questions of incidental abnormality detection, and systematic multi-architecture benchmarks have highlighted the need to report seed variance and paired comparisons in medical-imaging deep learning (33). Engaging this literature clarifies that the present results are promising but preliminary, and that the model should be viewed as an opportunistic screening aid on routine radiographs rather than a diagnostic substitute, pending external validation and prospective workflow evaluation.

Translating a model of this kind into practice requires more than high discriminative performance. A clinically useful screening aid must specify an operating point that reflects the intended referral pathway, report the resulting sensitivity, specificity, and expected referral volume, and be validated in the population and workflow in which it will be used (21, 26, 27). Because the cost of a missed case differs from the cost of an unnecessary DXA referral, threshold selection should be explicit and tied to local capacity and guidelines rather than fixed *a priori* (6, 7). Experience from opportunistic detection of vertebral fractures on routine computed tomography illustrates both the promise and the workflow challenges of this paradigm, including the need to define who acts on an incidental finding and how downstream confirmatory testing is organized (32). Comparable multi-architecture benchmarking studies also show that conclusions about model superiority are credible only when variability across seeds and splits is reported alongside paired comparisons (33).

This study has several limitations. First, it used a publicly available imaging dataset in which the original labels were assigned by the data provider according to radiographic features and were not uniformly verified against DXA-derived T-scores as the reference standard. The proposed model should therefore be regarded as a classifier of the dataset's radiographic labels rather than of DXA-defined bone mass status; its outputs are for radiographic reference only and cannot be equated with a clinical diagnosis of osteoporosis. Future studies should include real-world clinical data labeled using DXA as the reference standard to further validate and optimize performance. Second, the data partition was performed at the image level on an aggregated public collection; a perceptual-hash (pHash) de-duplication audit was performed and detected no exact duplicates, but because patient-level identifiers were not available, patient-level separation could not be performed, so possible leakage from near-duplicate images or multiple radiographs of the same patient cannot be excluded; a single random split and a single seed (42) were used, and because of limited computational resources repeated runs over multiple seeds and k-fold cross-validation could not be performed; the small test set (195 images, 38 osteopenia) with overlapping confidence intervals means that the observed differences should be interpreted with caution. Third, probability calibration and operating-point analysis were not reported, and no external multicenter validation, radiologist comparison, non-DXA-reference standard, or prospective workflow evaluation was performed. Fourth, the current preprocessing pipeline resized the entire image without knee region-of-interest segmentation, leaving potential bias from background content and acquisition quality. These factors limit the strength of evidence for translation into clinical practice and should be addressed in future work.

## 5 Conclusion

We developed and internally validated a multistage CBAM-enhanced model that classified the radiographically derived bone-mass labels (normal bone mass, osteopenia, and osteoporosis) of a public knee radiograph dataset. Within a single stratified split, the model achieved high accuracy and AUCs on the internal test set, and the ablation experiments and Grad-CAM analysis supported the effectiveness of the architecture and a degree of anatomical plausibility. This approach provides a feasible technical pathway for opportunistic screening of abnormal bone mass using routine radiographs. Before clinical implementation, however, DXA-paired labels, patient-level data partitioning with a de-duplication audit, repeated runs with paired statistical comparisons, probability calibration, multicenter external validation, and prospective workflow evaluation are required.

## Data Availability

The original contributions presented in the study are included in the article/Supplementary Material, further inquiries can be directed to the corresponding author.
